# Organizational Factors, Beliefs, Caring Behavior, and Quality of Nursing Work Life Among ICU Nurses: A Carolina Care–Informed Structural Model

**DOI:** 10.1155/nrp/6830564

**Published:** 2026-09-01

**Authors:** Tintin Sukartini, Yulis Setiya Dewi, Herdina Mariyanti, Vimala Ramoo, G. Kanchana, Muhammad Fikri Al-Faruq, Adinda Nurul Hamidah, Yumna Raihanatu, Alfi Syahri

**Affiliations:** ^1^ Faculty of Nursing, Universitas Airlangga, Surabaya, East Java, Indonesia, unair.ac.id; ^2^ Department of Advance Nursing and Research Group in Medical Surgical Nursing, Faculty of Nursing, Universitas Airlangga, Surabaya, Indonesia, unair.ac.id; ^3^ Department of Basic Nursing and Research Group in Medical Surgical Nursing, Faculty of Nursing, Universitas Airlangga, Surabaya, Indonesia, unair.ac.id; ^4^ School of Nursing, Faculty of Health Sciences, Nursing and Education, MAHSA University, Petaling Jaya, Malaysia, mahsa.edu.my; ^5^ Apollo College of Nursing, The Tamil Nadu Dr. M.G.R. Medical University, Chennai, India, tnmgrmu.ac.in; ^6^ Faculty of Nursing, Institut Kesehatan Deli Husada Deli Tua, Deli Serdang, North Sumatra, Indonesia; ^7^ Indonesia Palliative Nurses Association, Jakarta, Indonesia

**Keywords:** Belief, critical care, organizational culture, professional competence, quality of nursing work life, sustainable health workforce

## Abstract

**Background:**

The quality of nursing work life (QNWL) is essential to maintain nurses’ well‐being and professional performance, particularly in the intensive care unit (ICU) where workload, stress, and ethical challenges are high. Nurse characteristics, job demands, organizational support, Beliefs, and caring behavior are considered potential determinants of QNWL, but evidence regarding their pathways and mechanisms remains scarce.

**Aim:**

To analyze the effects of nurse‐related, job‐related, and organizational factors on nurses’ Beliefs, caring behavior, and QNWL among ICU nurses using a Carolina Care–based structural model, including the partial mediating roles of Beliefs and caring behavior.

**Study Design:**

A cross‐sectional design was applied to 200 ICU nurses from four hospitals. Data were collected using validated questionnaires assessing nurse characteristics, job factors, organizational support, Beliefs, and caring behavior, and QNWL PLS‐SEM was employed to evaluate convergent validity, construct reliability, and the inner model.

**Results:**

Nurse factors had a significant negative influence on Beliefs (*β* = −0.208; *p* < 0.001), whereas organizational factors strongly and positively affected both Beliefs (*β* = 0.534; *p* < 0.001) and caring behavior (*β* = 0.333; *p* < 0.001). Beliefs significantly influenced caring behavior (*β* = 0.242; *p* = 0.001), while caring behavior strongly predicted QNWL (*β* = 0.533; *p* < 0.001). Job factors did not significantly affect Beliefs (*β* = 0.067; *p* = 0.390), which was described as nonsignificant. The measurement model showed acceptable convergent validity and composite reliability for the main constructs, although the Beliefs construct was modeled using a single global indicator and the job factor construct showed Cronbach’s alpha slightly below the conventional threshold. The evaluation of the structural model showed that the endogenous variable had an *R* ^2^ value of 0.284, indicating a relatively modest level of explained variance. In addition, the *Q* ^2^ value of 0.219 suggests that the model demonstrates an acceptable level of predictive relevance.

**Conclusions:**

Organizational factors were the strongest predictors of Beliefs and caring behavior, both of which significantly improved QNWL. Supportive leadership, institutional policies, and recognition systems play key roles in shaping nurses’ Beliefs and behaviors. Organizational support emerges as the most powerful level for change, directly leading to the practice recommendations. Implications for clinical practice include strengthening organizational support, providing structured training, and enhancing recognition programs to improve QNWL and sustain a resilient, caring nursing workforce.

**Relevance to Clinical Practice:**

This study highlights that improving ICU nurses’ QNWL depends largely on strengthening organizational support, including supportive leadership, clear policies, and meaningful recognition. By fostering nurses’ Beliefs and caring behavior through structured education, mentoring, and a caring‐based practice environment, hospitals can enhance QNWL and sustain a resilient, compassionate ICU nursing workforce.

## 1. Introduction

The intensive care unit (ICU) is a highly complex and demanding clinical environment where nurses are required to provide continuous monitoring, rapid clinical decision‐making, advanced technical care, and emotional support for patients and families. ICU nurses frequently encounter high patient acuity, unstable clinical conditions, heavy workloads, time pressure, ethical dilemmas, and emotionally challenging situations, all of which may affect their professional performance and QNWL. In addition to physical workload, ICU nurses also experience psychological and emotional burdens due to repeated exposure to critical illness, patient deterioration, death, and family distress. These challenges are particularly relevant in hospital settings where staffing adequacy, organizational support, leadership, reward systems, and structured professional development may vary. Therefore, understanding the factors that shape ICU nurses’ Beliefs, caring behavior, and QNWL is essential for developing a supportive practice environment that sustains nurses’ well‐being and strengthens the quality of care delivered in critical care settings [1, 2].

Although previous studies have identified associations between nurse‐related, job‐related, and organizational factors and QNWL, no structural model has yet comprehensively integrated all these factors with caring Beliefs and behaviors in the context of intensive care. This study aims to analyze the effects of nurse‐related, job‐related, and organizational factors on nurses’ Beliefs, caring behavior, and QNWL among ICU nurses using a Carolina Care–based structural model, as well as to examine the mediating roles of nurses’ Beliefs and caring behavior.

The phenomenon of care practices in the ICU shows that many nurses still primarily base their interventions on medical–biological approaches, with insufficient involvement of the psychosocial and spiritual dimensions of care. This issue is directly related to the lack of adequate training and the absence of integration between nursing education curricula and a value‐based palliative care approach [3]. This limitation is further exacerbated by the challenges nurses face in providing the spiritual care that patients need during the end‐of‐life phase, especially in the context of Indonesian culture, where religious values are a critical foundation for healing and coping with death. The Carolina Care model (CCM) offers a conceptual solution to overcome this challenge through an approach that emphasizes improving communication competence, instilling professional ethics, and internalizing caring values in nursing practice [4, 5]. Nurses in the Carolina Care framework are encouraged to become facilitators of communication and protectors of human values, rather than simply implementers of medical actions.

## 2. Background

Nurses’ caring behavior is closely linked to improvements in QNWL and has a positive impact on job satisfaction and nurse retention [6–9]. A supportive work environment, effective clinical supervision, and training that strengthens emotional intelligence have been shown to encourage nurses to demonstrate consistent caring behavior. Organizational support and participatory leadership are key factors in fostering a work culture that prioritizes the values of empathy and spirituality in patient care, as suggested by several previous studies [1, 10].

The quality of life of nurses working in ICUs is significantly influenced by a supportive work environment, which in turn contributes to nurses’ work–life quality, [11]. Nurses’ self‐concept also has a significant influence on clinical decision‐making, which is directly related to caring behavior toward patients [12]. Furthermore, professional values are closely linked to nurses’ caring behavior, which plays a crucial role in improving the quality of patient care in the ICU [11, 13].

A positive work environment in the neonatal care unit is closely linked to nurses’ professional quality of life, a finding that is also relevant in other care units [13]. This study reinforces the established link between the organizational environment and professional well‐being (ProQoL) in critical care settings. Professional values serve as mediators in influencing nurses’ caring behavior in the ICU, where organizational factors contribute to improved caring behavior [14].

While there is substantial literature exploring the relationship between organizational factors and QNWL, there has been no structural model that comprehensively integrates these elements, including caring Beliefs and behaviors, for empirical testing in the ICU. Previous models have either focused on isolated constructs or have not sufficiently accounted for the complex interplay of individual, job‐related, and organizational factors. The absence of a comprehensive structural model that integrates nurses’ Beliefs and behaviors with QNWL in the context of intensive care is a significant research gap, particularly in health systems in developing countries.

This study aims to fill that gap by developing and validating a Carolina Care–based structural model in the ICU, using Structural Equation Modeling (SEM). This approach allows for the simultaneous testing of causal relationships between nurses′ Beliefs, caring behavior, and QNWL. The CCM, which emphasizes communication, human values, and the facilitative role of nurses, provides the theoretical foundation for this model. Through this lens, nurses are viewed as active facilitators of communication and protectors of human values, rather than just implementers of medical interventions.

The Health Belief Model (HBM), which operationalizes the construct of Beliefs, is used to explain how perceived vulnerability, severity, benefits, and barriers influence the nurses’ caring behavior. By integrating these two models, this study provides a comprehensive framework for understanding how organizational support, leadership, and reward systems shape nurses′ Beliefs and caring behavior, which in turn influence their QNWL.

The uniqueness of this model lies in its ability to integrate theoretical approaches (CCM and HBM), professional values, and empirical validation into one framework aimed at fostering excellence in practice. This model not only contributes to the development of palliative care systems but also represents an original contribution to the literature on nursing models in critical care, particularly in developing country contexts.

## 3. Aims and Hypotheses

**FIGURE 1:**
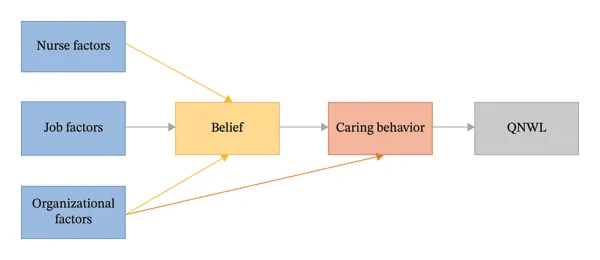
Hypothesized structural model of Carolina Care–based palliative nursing in the ICU. Conceptual model illustrating the proposed relationships between nurse factors, job factors, organizational factors, nurses’ Beliefs, caring behavior, and QNWL. Nurse, job, and organizational factors are hypothesized to influence Beliefs; organizational factors are also hypothesized to directly influence caring behavior; Beliefs are hypothesized to affect caring behavior, which in turn predicts QNWL.

This study aims to examine the relationships among nurse‐related factors, job‐related factors, organizational factors, nurses’ Beliefs, caring behavior, and QNWL among ICU nurses within a Carolina Care–based structural model, as illustrated in the hypothesized model (Figure 1). Specifically, this study evaluates whether nurses’ Beliefs and caring behavior function as partial mediators, rather than full mediators, in the pathways linking organizational factors to caring behavior and QNWL. Partial mediation was hypothesized because the model retains theoretically justified direct pathways, particularly from organizational factors to caring behavior, while also testing indirect pathways through nurses’ Beliefs and caring behavior.

 H1. Nurse‐related factors (age, gender, length of work, career‐level training, education, and employment status) significantly influence nurses’ Beliefs in providing palliative care.
 H2. Job‐related factors (workload, working conditions, and job design) significantly influence nurses’ Beliefs in providing palliative care.
 H3. Organizational factors (organizational support, leadership style, and reward systems) significantly influence nurses’ Beliefs in providing palliative care.
 H4. Organizational factors (organizational support, leadership style, and reward systems) significantly influence nurses’ caring behavior.
 H5. Nurses’ Beliefs, operationalized through their perceptions of vulnerability, severity, benefits, and barriers, significantly influence their caring behavior.
 H6. Nurses’ caring behavior, defined as compassion, maintenance of Beliefs, and professional competence, significantly influences QNWL.
 H7. Nurses’ Beliefs partially mediate the relationship between organizational factors and caring behavior.
 H8. Caring behavior partially mediates the relationship between organizational factors and QNWL.

## 4. Design and Methods

A quantitative cross‐sectional design was used to develop and test a Carolina Care–based structural model of palliative nursing in the ICU. Data were analyzed using partial least squares–structural equation modeling (PLS‐SEM) to examine the direct and mediated effects of nurse‐related, job‐related, and organizational factors on Beliefs, caring behavior, and QNWL. PLS‐SEM was chosen due to its suitability for testing complex models with multiple constructs and its ability to focus on prediction, making it ideal for examining both direct and indirect effects of nurse‐related, job‐related, and organizational factors on QNWL.

### 4.1. Setting and Sample

The study was conducted in the ICUs of four tertiary referral hospitals in Indonesia: Haji Regional General Hospital of East Java Province, Dr. Kariadi General Hospital Semarang, Sultan Agung Islamic Hospital Semarang, and Universitas Airlangga Hospital. The target population comprised registered nurses providing direct care in these ICUs. Participants were selected using purposive sampling with the following inclusion criteria: at least 1 year of ICU experience, a minimum educational level of diploma in nursing, active involvement in direct ICU care, and voluntary participation with written informed consent.

A total of 200 ICU nurses were included in the final analysis. This sample size was considered adequate for PLS‐SEM because the highest number of predictors directed to one endogenous construct in the model was three. Based on commonly applied PLS‐SEM sample‐size guidance and a priori power considerations for a three‐predictor model with 0.05 significance level, 0.80 power, and medium effect size, the required minimum sample was below 200. Therefore, the final sample was sufficient to estimate the proposed Carolina Care–based structural model and test the hypothesized direct and partial mediating effects.

### 4.2. Data Collection Tools and Methods

Data were collected using a self‐administered questionnaire package covering six main constructs: nurse factors, job factors, organizational factors, Beliefs, caring behavior, and QNWL. Nurse factors were obtained from demographic items (age, gender, ICU experience, employment status, and career level). Job factors included workload, working conditions, and job design, whereas organizational factors captured perceived organizational support, leadership style, and reward system. Nurses’ professional Beliefs regarding palliative care were operationalized using a single‐item global indicator, conceptually derived from the core tenets of the HBM (covering the themes of perceived vulnerability, severity, benefits, and barriers). Caring behavior comprised compassion, maintaining Belief, and professional competence, and QNWL encompassed work–life/home life, work design, work context, and work world dimensions.

The scales used in this study were adapted from validated instruments such as the “Caring Behavior Inventory” and “Nursing Work Index.”

Sample items for each key construct include the following:

1. Caring behavior: “I feel compassion for my patients when they are in distress.”
2. Job factors: “I am satisfied with my workload in the ICU.”
3. Organizational factors: “I feel supported by my organization in my role as a nurse.”
4. Beliefs: “I believe that palliative care is essential for improving the quality of life of terminal patients.”

The adaptation process involved translation and contextual modification to fit the ICU setting in Indonesia. Content validity was established through expert panel review, where a panel of three content experts in nursing and palliative care evaluated the clarity, relevance, and appropriateness of each item in the context of ICU nursing practice. All instruments were reviewed by content experts, and construct validity and reliability were evaluated in this sample. Questionnaires were distributed in person after written informed consent had been obtained and completed during work breaks under the supervision of the research team. A justification for collapsing Belief dimensions into a single indicator was provided to simplify measurement and ensure reliable construct validity, supported by strong convergent validity indicators.

### 4.3. Data Analysis

Data were entered and checked for completeness, then analyzed using descriptive statistics to summarize participants’ characteristics and study variables. PLS‐SEM was applied to assess the measurement model and the structural model. The first stage involved testing the measurement model to ensure that the constructs were valid and reliable. Convergent validity was evaluated using factor loadings (> 0.60) and Average Variance Extracted (AVE > 0.50), while construct reliability was examined using Cronbach’s alpha and composite reliability (CR) (> 0.70). Discriminant validity was confirmed using the Fornell–Larcker criterion and the Heterotrait–Monotrait ratio (HTMT). The second stage involved testing the structural model, where structural paths between latent variables were tested using bootstrapping to obtain path coefficients (*β*), t‐statistics, and *p* values. Bootstrapping was conducted with 5000 subsamples to generate the *t*‐statistics and *p* values, with *p* < 0.05 considered statistically significant.

## 5. Results

### 5.1. Participants

A total of 200 ICU nurses participated in this study. The majority of respondents were in the young adult age group of 20–29 years, comprising 168 nurses (84.0%), while 32 nurses (16.0%) were in the adult age group of 30–59 years. The mean age was 29.64 years (SD = 6.15; range = 25–57 years). This age distribution indicates that the sample was predominantly composed of relatively young ICU nurses. Therefore, the interpretation of nurse‐related factors, such as career level and length of ICU experience, should be considered within the context of an early‐ to mid‐career nursing workforce and should be generalized cautiously to older or more experienced ICU nurses. Most of the participants were female (54.0%), and more than half had a diploma in nursing (51.0%). The average work experience in ICU was 7.45 years (SD = 5.90), with 42.5% having 1–5 years of experience. More than half were civil servants (53.5%), and the most common career level was clinical nurse III (39.5%).

It is important to note that the study required a minimum of 1 year of ICU experience for inclusion, though a minimum of 0 years was reported for some participants, which is likely a data entry error. The full characteristics of the respondents are shown in Table 1.

**TABLE 1:** Distribution of respondent characteristics (*N* = 200).

Characteristics of respondents	n (%)	Mean ± SD (Min–Max)
Age		29.64 ± 6.15 (25.00–57.00)
Young adult (20–29 years)	168 (84.0)	
Adult (30–59 years)	32 (16.0)	
Total	200 (100.0)	
Gender		
Male	92 (46.0)	
Female	108 (54.0)	
Total	200 (100.0)	
Educational background		
Diploma in nursing (D3)	102 (51.0)	
Professional nurse (Ners)	91 (45.5)	
Master’s/specialist	7 (3.5)	
Total	200 (100.0)	
Years of working experience in ICU		
1–5 years	85 (42.5)	7.45 ± 5.90 (1.00–40.00)
5–10 years	68 (34.0)	
11–20 years	40 (20.0)	
> 20 years	7 (3.5)	
Total	200 (100.0)	
Employment status		
Regional public service agency	40 (20.0)	
Honorary/contract	53 (26.5)	
Civil servant	107 (53.5)	
Total	200 (100.0)	
Career level		
Clinical nurse I	46 (23.0)	
Clinical nurse II	73 (36.5)	
Clinical nurse III	79 (39.5)	
Clinical nurse IV	2 (1.0)	
Total	200 (100.0)	

*Note:* Sociodemographic and professional characteristics of ICU nurse participants, including age group, gender, educational background, years of ICU experience, employment status, and clinical career level. Data are presented as n (%) and mean ± SD (min–max).

### 5.2. Convergent and Construct Validity Results

The convergent validity test results in Table 2 show that most indicators have a loading factor value above 0.60, which generally indicates validity and significance in measuring their constructs. The Belief construct was evaluated using a single global indicator, resulting in a localized factor loading and an AVE value of 1.000. In alignment with PLS‐SEM guidelines for predictive modeling, single‐item constructs are statistically acceptable when the underlying concept is distinct and easily understood by respondents, though it shifts the model focus toward global predictive relevance rather than multidimensional scale validation.

**TABLE 2:** Convergent validity test results of the measurement model.

Serial number	Latent variables	Indicators/Manifest variables	Loading factor	Notes
1	Nurse factors (X1)	X1.1 Age	0.737	Valid/significant
		X1.2 Gender	0.624	Valid/significant
		X1.4 Length of work	0.660	Valid/significant
		X1.6 Career level	0.839	Valid/significant

2	Job factors (X2)	X2.1 Workload	0.854	Valid/significant
		X2.2 Working conditions	0.862	Valid/significant
		X2.3 Job design	0.607	Valid/significant

3	Organizational factors (X3)	X3.1 Organizational support	0.861	Valid/significant
		X3.2 Leadership style	0.848	Valid/significant
		X3.3 Reward system	0.737	Valid/significant

4	Beliefs (X4)	X4.1 Belief	1.000	Valid/significant

5	Caring behavior (X5)	X5.1 Compassion	0.904	Valid/significant
		X5.2 Maintaining belief	0.952	Valid/significant
		X5.3 Competence	0.944	Valid/significant

6	Quality of nursing work life (Y1)	Y1.1 Work life–home life dimensions	0.897	Valid/significant
		Y1.2 Work design dimensions	0.884	Valid/significant
		Y1.3 Work context dimensions	0.903	Valid/significant
		Y1.4 Work world dimensions	0.881	Valid/significant

*Note:* Factor loadings for all indicators of nurse factors (X1), job factors (X2), organizational factors (X3), Beliefs (X4), caring behavior (X5), and quality of nursing work life (Y1). All loading factors are > 0.60 and statistically significant, indicating acceptable convergent validity.

The nurse factors, work factors, organizational factors, Beliefs, caring behavior, and QNWL indicators all meet the validity criteria with values ranging from 0.607 to 0.952. The job design indicator demonstrated a loading factor of 0.607. Although this is closer to the lower boundary of the acceptable range, it was retained within the measurement model to preserve the conceptual coherence and content validity of the job factors construct, satisfying the minimum structural threshold of 0.60. As displayed in Table 3, the AVE values for all multiitem latent variables exceeded the standard 0.50 threshold, establishing sufficient convergent validity. The “Beliefs” construct registered an AVE value of 1.000, which is the mathematically expected output for a latent variable operationalized via a single‐item global proxy, thereby completing the measurement model requirements.

**TABLE 3:** Construct validity based on average variance extracted (AVE).

Variables	AVE values	Notes
X1 Nurse factors	0.518	Valid
X2 Job factors	0.614	Valid
X3 Organizational factors	0.668	Valid
X4 Beliefs	1.000	Valid
X5 Caring behavior	0.872	Valid
Y1 Quality of nursing work life	0.794	Valid

*Note:* AVE values for each latent variable (X1–X5 and Y1), showing that all constructs meet the criterion AVE ≥ 0.50 and thus demonstrate adequate convergent validity.

Abbreviation: AVE, average variance extracted.

### 5.3. Construct Reliability Test Results

The reliability test results in Table 4 show that all research variables meet the reliability criteria with Cronbach’s Alpha and CR values above 0.70. The nurse factor (*α* = 0.711; CR = 0.809), job factor (*α* = 0.676; CR = 0.823), and organizational factor (*α* = 0.752; CR = 0.857) have good internal consistency. The job factor construct registered Cronbach’s alpha of 0.676. While this falls slightly below the conventional 0.70 threshold, the construct’s CR was strong at 0.823, which firmly establishes its internal consistency and reliability for structural model testing. The Beliefs (*α* = 0.926; CR = 0.953) and caring behavior (*α* = 0.914; CR = 0.939) showed very high reliability, while QNWL (*α* = 0.704; CR = 0.818) was also recorded as reliable.

**TABLE 4:** Construct reliability test results.

Variables	Cronbach’s alpha	Composite reliability
X1 Nurse factors	0.711	0.809
X2 Job factors	0.676	0.823
X3 Organizational factors	0.752	0.857
X4 Beliefs	0.926	0.953
X5 Caring behavior	0.914	0.939
Y1 Quality of nursing work life	0.704	0.818

*Note:* Cronbach’s alpha and composite reliability coefficients for nurse factors, job factors, organizational factors, Beliefs, caring behavior, and quality of nursing work life, all above 0.70, indicating good internal consistency for each latent construct.

### 5.4. Structural Model Test Results (Inner Model)

The results of path analysis in Table 5 show different relationships between exogenous and endogenous latent variables. The nurse factor (X1) has a significant negative effect on Belief (X4) (*β* = −0.208; T = 3.813; and *p* < 0.001), while the caring behavior (X5) shows no significant effect (*β* = 0.042; T = 0.632; and *p* = 0.527). Work factors (X2) had no significant effect on Belief (X4) (*β* = 0.067; T = 0.859; and *p* = 0.390), and this result is described as nonsignificant for immediate clarity. The Beliefs variable was operationalized using a single observed item. While latent constructs are typically measured with multiple indicators to enhance robustness, a single‐item measure can be deemed appropriate when the concept is straightforward, unidimensional, and can be sufficiently captured through an overall or global assessment. Within the context of PLS‐SEM, this practice remains acceptable, particularly in research that emphasizes predictive modeling rather than the development or validation of measurement scales [15, 16].

**TABLE 5:** Path coefficients and *t*‐test results for exogenous to endogenous latent variables (inner model).

Variable	Original sample	Sample mean	SD	T statistics	*p* value	Notes
X1 ⟶ X4 (Nurse ⟶ Belief)	−0.208	−0.208	0.054	3.813	0.001	Significant
X1 ⟶ X5 (Nurse ⟶ Caring)	0.042	0.037	0.066	0.632	0.527	Not significant
X2 ⟶ X4 (Job ⟶ Belief)	0.067	0.074	0.078	0.859	0.390	Not significant
X3 ⟶ X4 (Organization ⟶ Belief)	0.534	0.534	0.074	7.227	0.000	Significant
X3 ⟶ X5 (Organization ⟶ Caring)	0.333	0.339	0.073	4.587	0.001	Significant
X4 ⟶ X (Belief ⟶ Caring)	0.242	0.236	0.074	3.263	0.001	Significant
X5 ⟶ Y1 (Caring ⟶ QoNWL)	0.533	0.533	0.056	9.538	0.001	Significant

*Note:* Partial least squares–structural equation modeling results showing original sample estimates, sample means, standard deviations, *t* statistics, and p values for the structural paths between nurse factors (X1), job factors (X2), organizational factors (X3), Beliefs (X4), caring behavior (X5), and quality of nursing work life (Y1). Paths with *p* < 0.05 are considered statistically significant.

Abbreviations: QNWL, quality of nursing work life; PLS‐SEM, partial least squares–structural equation modeling; SD, standard deviation.

In contrast, organizational factors (X3) had a strong and significant positive effect on Beliefs (X4) (*β* = 0.534; T = 7.227; and *p* < 0.001) as well as on caring behavior (X5) (*β* = 0.333; T = 4.587; and *p* < 0.001). Belief (X4) significantly influenced caring behavior (X5) (*β* = 0.242; T = 3.263; and *p* < 0.001). Caring behavior (X5) was shown to have a highly significant positive influence on QNWL (Y1) (*β* = 0.533; T = 9.538; and *p* < 0.001) (The evaluation of the structural model showed that the endogenous variable had an *R* ^2^ value of 0.284, indicating a relatively modest level of explained variance. In addition, the *Q* ^2^ value of 0.219 suggests that the model demonstrates an acceptable level of predictive relevance).

**FIGURE 2:**
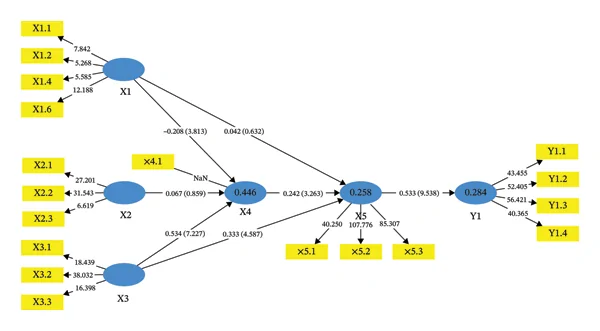
Final structural model with standardized path coefficients. Partial Least Squares–Structural Equation Model showing the significant paths between nurse factors (X1), job factors (X2), organizational factors (X3), Beliefs (X4), caring behavior (X5), and quality of nursing work life (Y1). Ovals represent latent variables, rectangles represent observed indicators, and arrows represent directional relationships. Numbers on the paths indicate standardized path coefficients (*β*) with t statistics in parentheses; only statistically significant paths are highlighted.

The complete visualization of the structural model test results, including path coefficients and significance values, can be seen in Figure 2, where nonsignificant paths are visually distinguished with dashed lines or explicitly noted in the figure caption for better interpretability.

## 6. Discussion

This study aims to develop and validate a structural model of palliative nursing in the ICU by referring to the principles of the CCM. The model was built on six main constructs, namely, nurse factors, work factors, organizational factors, Beliefs, caring behavior, and QNWL, then tested for interrelationships using SEM.

The research findings show that caregiver factors actually have a negative effect on Beliefs. Demographic characteristics, such as age, gender, education level, and career level, which are often assumed to strengthen Beliefs, did not consistently have a positive impact. Exposure to stress, emotional exhaustion, and moral distress in nurses with long experience and high career positions has the potential to weaken Beliefs in palliative practice. This finding can be linked to concepts like moral distress, compassion fatigue, or resilience depletion, particularly in ICU settings where long‐term exposure to stress may take a psychological toll on nurses. The resilience depletion and chronic stress in ICU nurses can negatively impact their coping abilities and emotional responses, which could explain why experienced nurses may exhibit diminished Beliefs despite their professional competence. This suggests that strengthening Beliefs in palliative care requires ongoing organizational support and training rather than relying solely on demographic factors [17, 18].

Analysis of Job factors showed that this variable did not have a significant effect on Beliefs. While previous literature has emphasized the role of workload and working conditions in nursing practice, this finding suggests that Belief systems are more supported by moral values and organizational culture. The HBM suggests that Beliefs are more strongly influenced by perceived environmental cues and the value‐based organizational culture rather than the immediate stressors of daily tasks. For instance, workload may not directly impact Beliefs, but a supportive organizational environment can promote a culture where Beliefs are aligned with ethical values and professional standards, thus strengthening nurses’ commitment to palliative care [19]. Workload may not directly affect Beliefs, but a supportive organizational culture, as suggested by the HBM, may help shape Beliefs by providing cues to action that are more aligned with ethical values than task‐related stressors.

Organizational factors emerged as the strongest predictors of both caring Beliefs and behaviors. Supportive leadership within the organizational context facilitates the internalization of caring values (CCM) and reduces perceived barriers to providing palliative care (HBM). Reference [20] showed that the professional practice environment is directly correlated with nursing outcomes. Reference [1] emphasized the need for managerial prerequisites in palliative integration, while reference [4] highlighted the role of palliative care nurse champions as agents of organizational culture transformation. Reference [21] added the importance of informal leaders as drivers of practice. All of this evidence reinforces the relevance of CCM, which places organizational support at the core of shaping values, Beliefs, and caring behaviors in critical care settings. Supportive leadership acts as a bridge to facilitate the internalization of caring values, which in turn reduces perceived barriers to providing palliative care, aligning with the HBM’s focus on perceived barriers and cues to action [4].

Belief has been shown to have a significant effect on caring behavior and acts as an important mediator in nursing practice. The dimensions of perceived vulnerability, severity, benefits, and barriers shape the expression of compassion, the maintenance of patient Beliefs, and the display of nurses’ professional competence. Bakir and Sü [22] emphasized professional values as predictors of caring attitudes, while Seyedfatemi et al. [23] linked caring behavior with death anxiety where ethical Beliefs serve as a protective barrier. Reference [24] also showed that value orientation and personal Beliefs influence nurses’ perceptions of early palliative care. This confirms the role of Beliefs as a moral foundation that reinforces caring behavior.

Caring behavior is further closely related to QNWL. Caring is not only a core value of the profession but also a determinant of nurses’ professional well‐being. QNWL dimensions including work–life balance, work context, satisfaction, and professional support increase with the implementation of good caring behaviors. Arsat et al. [6] found a link between caring behaviors and a supportive work environment, while Xiao et al. [9] emphasized its relationship with job satisfaction as well as psychological factors. Ocalan et al. [25] proved that spirituality and caring values con[9]tribute significantly to the quality of nursing work life (QNWL). These findings show that caring behavior is not only an ethical obligation but also the foundation of professional sustainability as well as the key to increasing job satisfaction.

The structural model demonstrates that nurses’ Beliefs exert a significant positive effect on their clinical caring behavior (beta = 0.242, *p* = 0.001$), acting as an essential moral mediator. By utilizing a comprehensive global indicator, this construct successfully capsulized the intrinsic value orientation rooted in the HBM frameworks. While multiitem latent scales are traditional for exploring fragmented behavioral dimensions, our deployment of a single‐item global proxy concentrated the measurement directly on the unified ethical conviction of the nurse. This moral foundation directly translates into enhanced expressions of compassion and professional competence in critical care.

Furthermore, because the current study operationalized the Belief construct through a single global indicator, future structural models should expand this variable into a multiitem, multidimensional scale that psychometrically breaks down and separates individual HBM sub‐constructs (such as separating distinct indicators for perceived barriers versus perceived benefits) to unlock deeper diagnostic nuances in critical care settings.

## 7. Limitations

This study has several limitations:

• Cross‐Sectional Design: The cross‐sectional nature of the study limits causal inference, as it only provides a snapshot of the relationships between variables at one point in time.
• Self‐Report Bias: The use of self‐report‐based questionnaires may introduce bias, as respondents may provide socially desirable answers or may not accurately recall experiences.
• Convenience/Purposive Sampling: Since purposive sampling was used, the sample may not be fully representative of the larger population of ICU nurses, even within the four hospitals, which could limit the generalizability of the findings.
• Common Method Variance (CMV): The reliance on a single self‐report questionnaire at one time point may lead to common method bias, inflating correlations between constructs. This should be considered when interpreting the findings.
• Context Specificity: The model was tested in Indonesian public hospitals, and its applicability to other settings, such as private hospitals or culturally distinct contexts (e.g., conflict zones with extreme stress), remains unknown. Future research should explore the model’s applicability in different healthcare settings and cultural contexts.
• The study did not collect data on the total number of eligible ICU nurses across the four hospitals, the number approached, the recruitment yield, the response rate, or the numbers who declined participation or were excluded. This limits the ability to fully assess potential selection bias and may restrict the external validity and generalizability of the findings beyond the study settings.

Future research should consider using multiple data sources or a longitudinal design to reduce potential bias and enhance the robustness of the results.

## 8. Recommendations for Practice and Further Research

The findings of this study highlight the central role of organizational factors and caring behavior in enhancing ICU QNWL, suggesting that hospitals should prioritize organizational strategies that foster a supportive, caring‐oriented practice environment. Nurse managers and hospital leaders are encouraged to strengthen organizational support through authentic and participatory leadership, fair and transparent reward systems, and policies that explicitly integrate palliative and holistic care into ICU standards of practice. Structured, ongoing education based on the CCM focusing on communication, compassion, maintenance of patient Belief, and professional competence should be embedded into in‐service training, clinical supervision, and mentoring programs. Routine reflective forums, case discussions, and debriefings related to end‐of‐life and palliative cases may help sustain nurses’ Beliefs and caring behavior and, in turn, improve their QNWL. Future research should focus on priority interventions such as leadership training programs to enhance supportive leadership styles and improve organizational culture, as well as enhanced reward systems to recognize and support ICU nurses, reinforcing their caring behavior and improving their quality of nursing work life. Furthermore, future research should test and refine the proposed structural model in different clinical settings (such as oncology, emergency, and general wards) and across diverse cultural contexts to enhance its generalizability. Longitudinal and intervention studies are needed to evaluate the causal impact of organizational changes, leadership development, and Carolina Care–based training programs on nurses’ Beliefs, caring behavior, QNWL, and patient outcomes. Mixed‐methods designs could provide deeper insight into how nurses experience organizational support, moral distress, and spiritual aspects of care in daily ICU practice, thereby informing the development of more nuanced and contextually relevant palliative nursing interventions.

## 9. Conclusion

This study developed a theoretical framework to examine the pathways of influence of nurse, work, and organizational factors on caring behavior and QNWL, with Beliefs as the mediating variable. Results showed that organizational factors emerged as the strongest predictors of Beliefs and caring behavior, while nurse factors had a negative effect on Beliefs, and job factors had no significant effect. Beliefs proved to be an important mediator of caring behaviors, while caring behaviors had the greatest positive effect on QNWL. These findings enrich nursing science by confirming the central role of organizational support, leadership, and reward systems in strengthening nurses’ caring Beliefs and practices, which in turn improve their QNWL. The practical implication is that hospitals need to prioritize organizational strategies such as developing supportive leadership, reward systems, and structured training in palliative and holistic care to improve ICU nurses’ caring behaviors and QNWL. Hospital administrators must invest in fostering a supportive organizational culture and strong leadership as the primary strategy to enhance nurses’ caring capacity and, consequently, their own QNWL. Further research is recommended to be extended to various healthcare contexts, using longitudinal or interventional designs, and incorporating cross‐cultural perspectives to validate and strengthen the proposed framework, thereby contributing to the development of resilient and caring‐oriented nursing practice.

## Author Contributions

Tintin Sukartini: conceptualization, supervision, and validation. Yulis Setiya Dewi: methodology, investigation, and project administration. Herdina Mariyanti: data curation and resources. Vimala Ramoo: formal analysis, review, and editing. G. Kanchana: visualization and validation. Muhammad Fikri Al‐Faruq: software and data curation. Adinda Nurul Hamidah: investigation and resources. Yumna Raihanatu: investigation and visualization. Alfi Syahri: original draft preparation and methodology. All authors contributed to the manuscript and reviewed the final version.

## Funding

This study wassupported by the Universitas Airlangga International Research Consortium Scheme of 2024, pursuant to Rector’s Decree Contract No. 177/UN3.LPPM/PT.01.03/2024.

## Disclosure

The manuscript is approved by all authors for publication. The funder had no role in the study design, data collection, data analysis, interpretation of findings, manuscript preparation, or decision to submit the article for publication

## Ethics Statement

This study has obtained ethical approval from the Ethics Committee of the Universitas Airlangga Hospital (Number: 131/KEP/2024). The purpose and procedures of the study were explained in detail in the informed consent form, including the voluntary nature of participation as well as the guarantee of respondents’ anonymity. All data were stored securely in a password‐protected computer and could only be accessed by the research team.

## Conflicts of Interest

The authors declare no conflicts of interest.

## Data Availability

The datasets are not publicly available due to participant confidentiality and institutional ethical restrictions. De‐identified data, the codebook, PLS‐SEM analysis syntax, and study instruments are available from the corresponding author upon reasonable request and with appropriate institutional approval.
